# Vision Transformer and Deep Sequence Learning for Human Activity Recognition in Surveillance Videos

**DOI:** 10.1155/2022/3454167

**Published:** 2022-04-04

**Authors:** Altaf Hussain, Tanveer Hussain, Waseem Ullah, Sung Wook Baik

**Affiliations:** Sejong University, Seoul 143-747, Republic of Korea

## Abstract

Human Activity Recognition is an active research area with several Convolutional Neural Network (CNN) based features extraction and classification methods employed for surveillance and other applications. However, accurate identification of HAR from a sequence of frames is a challenging task due to cluttered background, different viewpoints, low resolution, and partial occlusion. Current CNN-based techniques use large-scale computational classifiers along with convolutional operators having local receptive fields, limiting their performance to capture long-range temporal information. Therefore, in this work, we introduce a convolution-free approach for accurate HAR, which overcomes the above-mentioned problems and accurately encodes relative spatial information. In the proposed framework, the frame-level features are extracted via pretrained Vision Transformer; next, these features are passed to multilayer long short-term memory to capture the long-range dependencies of the actions in the surveillance videos. To validate the performance of the proposed framework, we carried out extensive experiments on UCF50 and HMDB51 benchmark HAR datasets and improved accuracy by 0.944% and 1.414%, respectively, when compared to state-of-the-art deep models.

## 1. Introduction

Nowadays, surveillance systems are increasingly installed for monitoring purposes to ensure public safety and put an effort to mitigate crimes [1, 2]. Therefore, enormous amounts of data are generated on the daily basis from the CCTV cameras, requiring manual monitoring for undesired activities [3]. It is almost impossible for human being to monitor multiple video streams, particularly, simultaneous inspection of several cameras for the identification of different activities and events. Therefore, automating the process of Human Activity Recognition (HAR) using image processing and deep learning techniques offers promising solution to this problem. Computer vision techniques for HAR in surveillance system are reliable sources for automatic decision-making, which are responsible for identifying individuals performing suspicious activities and informing the law enforcement agencies to take appropriate preemptive actions. Besides smart surveillance, HAR has numerous applications such as video retrieval [4] and video summarization [5]. However, accurate HAR using computer vision techniques is challenging due to instantaneous transition of events in successive frames, illumination variations, different viewpoints, cluttered background, and different scaling [6]. In videos context, activity recognition relies on the collection of multiple consecutive video frames, where both spatial and temporal information need to be analysed for an individual's body movements. The HAR related literature can be broadly divided into traditional features and deep learning-based techniques, as discussed in the subsequent sections. The traditional methods mainly consist of three steps: (1) preprocessing step is used to remove outliers and noise, (2) the preprocessed data undergo several low-level features extraction phases, and (3) in classification step, the extracted features are intelligently mapped with their corresponding classes. Since our model is based on deep features, therefore, herein, we only discuss deep features-based methods, but interested readers are referred to extensive reviews of baseline HAR methods [7, 8].

*Deep Learning-Based Techniques*. Considering the limited performance of traditional features and machine learning methods in HAR domain, researchers focus on deep learning-based techniques that process data in an end-to-end manner for features extraction and classification. CNN-based techniques extract features in a hierarchical way, where initial layers extract local features and the final layers extract global features. The standard 2D CNN is effective in learning the spatial information but is unable to learn the temporal information, which is important for improved accuracy in the case of HAR. For instance, Karpathy et al. [9] used pretrained CNN model which fuses the information from multiple frames for efficient activity recognition. This work is extended by Simmoyan and Zisserman [10], where the authors proposed a two-stream CNN network to overcome the issue of motion information involvement in HAR by employing optical flow features. But, in this method, the long-range contextual information is not considered, as the final activity prediction is generated by taking the average of predictions from a segmented video shot of variable length, that is, 10 to 15 frames sequence. Recently, simple 2D CNN architecture is widely employed in HAR literature; for instance, Khan et al. [11] used a lightweight CNN MobileNet model to cover violent activity scenes in the movies. Similarly, in another research, the Hough forests features are combined with 2D CNN to train a hybrid model for violent activity recognition [12]. The authors claim that their method requires less computation time, but due to complex poses, different scales, and illuminations, such methods are not effective in real-world situations. In this regard, the 2D CNN-based methods and motion representation attributes are gradually upgraded from 2D to 3D CNN, which considers both spatial and temporal information [13]. Researchers thus introduced 3D CNN and its variants to capture spatio-temporal information. In this direction, Tran et al. [14] proposed a 3D CNN (C3D) for the temporal and spatial features representation in the video data, outperforming the existing methods for HAR. In another research, Carreira et al. [15] proposed a novel mechanism where the pretrained ImageNet 2D filters are modified into their corresponding 3D version for activity recognition. The experiments concluded that their method achieved higher accuracy as compared to randomly initialized filters. Similarly, Hussain et al. [16] have proposed a lightweight 3D CNN model for anomaly activity recognition and camera prioritization in surveillance environments. The variants of 3D CNNs include two-stream 3D CNN [17], pseudo-3D CNN [18], and MiCT-Net [19]. However, the existing 3D-CNN models can only process 10 to 16 frames effectively. They cannot recognize lengthy activities due to exponential increase in time complexity caused mainly by the temporal dimension [16]. To overcome this issue, researchers experimented on hybrid models where the spatial features are extracted from pretrained CNN models and learned the temporal information using variants of Recurrent Neural Networks (RNNs).

The hybrid models extract frame-level features using CNNs or optical flow models, which are stacked using a predefined time stamp to learn temporal information that are fed to RNN variants such as long short-term memory (LSTM) Network [20] and Gated Recurrent Unit (GRU) [21] to improve the HAR performance. For sequence learning, the hybrid models have used RNN, LSTM, and GRU but without focusing on the selective information in the spatial domain from the consecutive sequences, which is very important to maintain connectivity between the frames. However, extracting selective features and discriminative information for HAR in complex surveillance environments [22] is a challenging task. Therefore, researchers proposed different techniques; for example, in an existing research, Li et al. [23] extracted C3D features from the input video sequence by using sliding window techniques to generate cubes, which are fed to LSTM network for HAR. Ma et al. [24] utilized VGG19 spatial features in their framework and employed multilayer LSTM for optimal activity recognition. The AlexNet CNN model is used in a baseline research [25] for spatial features extraction, and then deep bidirectional LSTM is used for temporal learning. The authors in [26] have proposed an efficient approach for real-time HAR in surveillance systems by using MobileNet architecture for spatial features extraction followed by sequential learning with LSTM. More recently, Hussain et al. [27] have achieved significant performance by integrating CNN features with support vector machine as sequential learning model, which is not considered to be more robust and generalized towards patterns learning when compared to recent deep sequential models.

In the existing methods, the kernels of the CNNs are mainly designed to capture short-range spatial-temporal information and they are limited to learn the long-range dependencies when they are beyond their receptive field. However, stacking convolutional layers [10, 13], naturally, extends the receptive field, but these strategies are inherently limited in capturing long-range dependencies by means of aggregation of shorter-range information. Still, the long-range temporal dependency is unresolved because the above-mentioned approaches are strongly relying on the weak features selection [27]. Similarly, capturing long-range sequences dependency is a major problem in different domains such as natural language processing (NLP) in machine translation [28, 29], autoregressive word generation [30], and question answering [31]. Therefore, the field of NLP has been revolutionized with the emerging technique such as self-attention or transformer [32]. Activity recognition and NLP share several high-level similarities; for example, sentences and videos both are sequential forms of data, where a single word is insufficient to understand its contextual meaning in terms of a sentence similar to a video where a single frame is not enough to understand semantics of the whole video. In a baseline research for Vision Transformer (ViT) [33], the authors extracted the local spatial features from an input image using patching strategy and then encoded these features to a standard transformer from the NLP with novel modifications; as a result, they achieved superior performance against state-of-the-art for classification tasks. Therefore, in this work, we investigated that long-range self-attention model would be highly effective in HAR. The problem of learning long-range spatiotemporal features in the HAR is resolved by discriminative spatial features extraction via ViT. Therefore, the frame-level features are extracted from the pretrained ViT-Base-16 followed by LSTM for the HAR. The main contributions of the proposed method are as follows:

1. We propose a novel mechanism that utilizes CNN-free approach to capture surveillance videos long-range temporal dependencies using ViT, followed by a sequential learning method to achieve new state-of-the-art accuracy when compared to existing HAR methods.
2. Spatial and temporal features play an important role in the accurate HAR, where we employ ViT for spatial features and multilayered LSTM to learn temporal relationships among these features to recognize human activities with higher confidence.
3. The performance of the proposed framework is evaluated on the challenging HMDB51 and UCF50 HAR datasets. The experimental results accomplished new state-of-the-art accuracy of 73.714% using HMDB51 and 96.144% accuracy using UCF50.

The rest of the paper is organized as follows: Section 2 presents the proposed activity recognition framework. Experimental setup, datasets, discussion on the results, and comparative analysis are given in Section 3. The conclusion and future works are given in Section 4.

## 2. The Proposed Activity Recognition Framework

**Figure 1:**
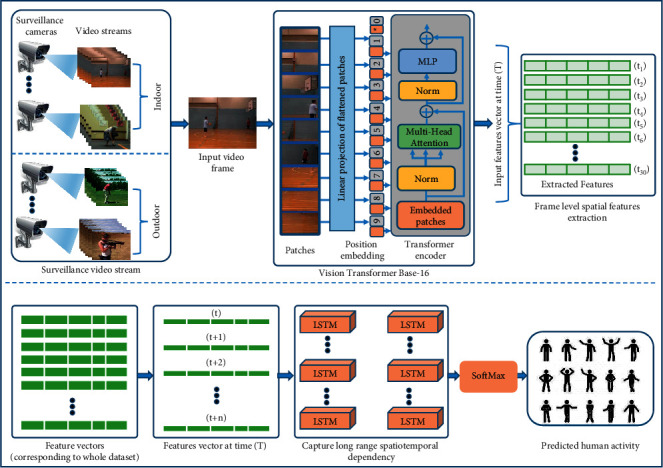
The proposed framework for automatic activity recognition in surveillance videos.

The proposed framework for HAR mainly consists of three steps, as visualized in Figure 1. In the first step, surveillance cameras capture video streams which are then fed to pretrained ViT-Base-16 for frame-level spatial features extraction. The spatial features are stacked together to create a resultant feature vector from 30 consecutive frames. In the third step, the generated feature vector is fed into a multilayer LSTM network to capture long-range spatial-temporal dependency in the HAR.

### 2.1. Features Extraction Using Vision Transformer

The architecture of ViT-Base-16 is entirely based on the standard transformer [32] architecture and achieved remarkable accuracy when compared to CNN-based models for image classification tasks. It uses self-attention mechanism to capture long-range relationship between input sequences. ViT is actually an attempt to use transformer model for image classification. Basically, it divides the input image into a number of patches that are linearly projected with learnable positional embedding to learn the order of patches followed by transformer encoder with multilayer perceptron for final classification.

In the first part, the input image is divided into nonoverlapping patches, because a standard transformer receives 1D sequence of token as an input. Usually, the image is in 2D format; therefore, to handle the 2D image, an image *x* ∈ ℝ^*H*×*W*×*C*^ is reshaped   into a sequence of flattened 2D patches *x*_*p*_ ∈ ℝ^*N*×(*P*^2^.*C*)^ . Herein, (*H*, *W*, *C*) represents the height, width, and channels of the image, while (*P*, *P*) is the resolution of each image patch, and (*N* = *HW*/*P*^2^) is the total number of patches. Typically, the patch size *P* is chosen as 16 × 16 *or* 32 × 32, where the small *P* size is able to capture longer sequences and vice versa. In our case, we have used the 16 × 16*P* for features extraction; in the subsequent section, these submodules are discussed in detail.

### 2.2. Linear Embedding Layer

The sequence patches are linearly projected into a vector with dimension *d* using a learn embedding matrix  *E*. Then, these embedded representations are concatenated together with learnable classification token *v*_*class*_. The embedded patches are without order; therefore, positional information *E*_*pos*_ is used to reorder the spatial information as the original image. The result of embedded patches with token  *Z*_0_ is mathematically represented in (1).

### 2.3. Vision Transformer Encoder

**Figure 2:**
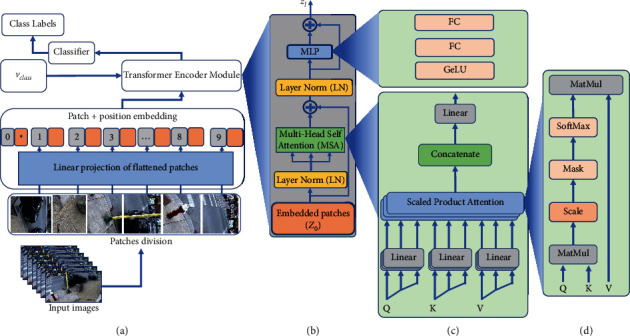
The Vision Transformer architecture: (a) the main architecture of the model, (b) the transformer encoder module, (c) multiscale self-attention (MSA) head, and (d) the self-attention (SA) head.

The resultant embedding patches of *Z*_0_ (in (1)) are fed to the transformer encoder module, which consists of *L* identical layers as shown in Figure 2. Furthermore, each module is divided into two components such as multihead self-attention (MSA) block and multilayer perceptron (MLP). The last block of MLP consists of two dense layers. Equations (2) and (3) represent the mathematical representations of the MSA and MLP, respectively.

(1) $$\begin{array}{c} Z_{0}=\left[v_{calss};x_{1}E;x_{2}E;\;..x_{n}E\right]+E_{pos},E\in {\mathbb{R}}^{\left(P^{2}.C\right)\times d},\;E\in {\mathbb{R}}^{\left(n+1\right)\times d}. \end{array}$$

(2) $$\begin{array}{c} z_{l}^{\prime }=MSA\left(LN\left(z_{l-1}\right)\right)+z_{l-1},\;l=1\ldots L, \end{array}$$

(3) $$\begin{array}{c} z_{l}=MLP\left(LN\left(z_{l}^{\prime }\right)\right)+z_{l}^{\prime },\;l=1\ldots L. \end{array}$$

In the last layer of the encoder, the first element *z*_*L*_^0^ in the sequence is passed to external head classifier for predicting the class label.

(4) $$\begin{array}{c} y=LN\left(z_{L}^{0}\right). \end{array}$$

The MSA is the central component of the transformer model which calculates the most and least important patch and discard the later one from the input sequence. It is further divided into four layers such as linear, self-attention, and concatenation layers to combine the output from the multiple heads, as their graphical representation is shown in Figure 2(c). Basically, the attention mechanism can be represented by attention weights which is calculated from the weighted sum of all values in sequences *z*. Three values, *Q* (query), *K* (key), and *V* (value), are calculated from the input sequence by multiplying the elements (Q, K) against three learning matrices U_*QKV*_; a single SA is graphically represented in Figure 2(d), while the mathematical formulation is given in equation (5):

(5) $$\begin{array}{c} \left[Q,K,V\right]=zU_{QKV},U_{QKV\;}\in {\mathbb{R}}^{d\times 3D_{K}}. \end{array}$$

In a given input sequence, to calculate the importance of one element with respect to others, the value of *Q* vector is multiplied by dot product with the *K* vectors. Then their result is scaled and passed to SoftMax activation function to find out the importance of patch with high attention score, as given mathematically in equation (6):

(6) $$\begin{array}{c} A=SoftMax\left(\frac{QK^{T}}{\sqrt{D_{K}}}\right),\;A\in {\mathbb{R}}^{n\times n}. \end{array}$$

The MSA is actually the combination of the multiple attention heads *h* instead of single values of *Q*, *K*, and *V*. For robust and optimal features selection, the results of each *SA* are concatenated and then projected through a feedforward layer with learnable weights *W* to the desired dimensions, as expressed in equation (7):

(7) $$\begin{array}{c} MSA\left(z\right)=Concat\left(SA_{1}\left(z\right);SA_{2}\left(z\right);\ldots SA_{h}\left(z\right)\right)W,W\in {\mathbb{R}}^{h.D_{K}\times D}. \end{array}$$

### 2.4. Learning Long-Range Temporal Dependencies via LSTM

Temporal features are very important to learn long-range dependencies in activity recognition. The RNN model is specifically designed for the time series or continuous data but recently researchers are inspired by their performance in activity recognition domain [34]. It combines the learned information from the previous and the current frames in an input video sequence for accurate HAR identification. However, the RNN is unable to hold long-range temporal dependencies due to vanishing gradient problem, which is solved by LSTM [35], that is capable of holding long-range temporal information. In the LSTM architecture, there are three gates, (1) input, (2) output, and (3) forget gates.  Table 1 shows the parameters details used to formulate the internal mechanism of LSTM to capture long-range temporal dependency for HAR.

The last gate f_t_ is responsible for retaining or discarding irrelevant information in input Δ*t* and from the previous output S_t−1_ [36, 37]. The frame-level discriminative features from the ViT at the unit time, *t*, are passed into the stacked LSTM network and f_t_ to hold long-term temporal dependency. Equations (8) to (14) show the mathematical representation of the LSTM network.

(8) $$\begin{array}{c} i_{t}=\partial \left(w_{i}\left[\Delta t+S_{t-1}\right]+b_{i}\right), \end{array}$$

(9) $$\begin{array}{c} f_{t}=\partial \left(w_{f}\left[\Delta t+S_{t-1}\right]+b_{f}\right), \end{array}$$

(10) $$\begin{array}{c} O_{t}=\partial \left(w_{0}\left[\Delta t+S_{t-1}\right]+b_{0}\right), \end{array}$$

(11) $$\begin{array}{c} R=\tanh\left(w_{R}\left[\Delta t+S_{t-1}\right]+b_{R}\right), \end{array}$$

(12) $$\begin{array}{c} C_{t}=C_{t-1}.f_{t}+R.i_{t}, \end{array}$$

(13) $$\begin{array}{c} S_{t}=tahn\left(C_{t}\right).\;O_{t}, \end{array}$$

(14) $$\begin{array}{c} \mathrm{Pr}ediction_{state}=soft\max\left(s_{t}N\right). \end{array}$$

Herein, the term Δ*t* represents the input over time and sigmoid activation function is represented by ∂. Their weights and bias terms are represented by *w* and *b*, respectively. The forget gate f_t_ at time *t* keeps the information of the previous frame that is needed and discard it otherwise. The output gate O_t_ keeps the information of the upcoming step and R is the recurrent unit having tanh activation function. It is computed from the input of the current frame and state of the previous frame S_t−1_. The RNN hidden state is calculated by the tanh activation and memory cell C_t_. The activity recognition does not need intermediate output from the LSTM; therefore, we use the SoftMax activation for final classification in (14), and N is used to represent the number of classes, that is, 51 for HMDB51 and 50 for UCF50 dataset.

### 2.5. Modeling Human Activity Recognition via ViT and Multilayer LSTM

Recently, ViT have dominated CNNs for image representation, leading to better classification [33] and segmentation results [38]. Herein, inspired by the better representation abilities of ViT, we extract spatial transformer features in our framework using pretrained ViT model, followed by sequential learning method to learn the temporal dependencies and interpretations of input frames. There are different variants of ViT models such as ViT-Base, ViT-Large, and ViT-Huge. The ViT-Base-16 achieved remarkable accuracy when compared to existing image classification methods using benchmark datasets, indicating its robust and representative features potentials. The ViT models have varied number of encoder layers, hidden dimension size, number of attention heads used by MSA layer, and MLP classifier size, as detailed in Table 2, where we employ ViT-Base-16 model with 16×16 patch size in the proposed framework.

Due to complex patterns of actions and temporal gaps between sequential actions merged together to form single activity, a single LSTM cell is unable to learn the patterns accurately. Therefore, we have conducted multiple experiments and stacked multiple LSTM cells to learn long-term temporal patterns across the video sequences.  Table 3 shows the proposed LSTM network to capture long-range temporal dependency of activity recognition. The ViT-Base-16 extracts 1000 features vector from each frame; therefore, our proposed sequential learning model takes 30 frames with 1000 spatial features vector.

Initially, the features vector contains enriched patterns information; therefore, we have used 128 LSTM units to learn all possible discriminative features. Then the features space is reduced by 64 numbers of LSTM units to efficiently map the number of classes, that is, 51 and 50 classes of HMDB51 and UCF50, respectively. Furthermore, to avoid overfitting and make the network more stable during training with faster learning, we utilized a 50% dropout and batch normalization. On top of this, we also performed experiments using different learning rates (LR) because it is one of the most important hyperparameters, which greatly affects the generalization of the model. When we take 1e-1 LR, the weight of the model is updated drastically and causes overshoot due to which the model does not reach global minima and is stuck in the local minima. After multiple experiments, when we take 1e-4 LR, the model achieves the highest accuracy of 73.714% and 96.144% on the HMDB51 and UCF50 datasets, respectively.

## 3. Experimental Results and Discussion

**Figure 3:**
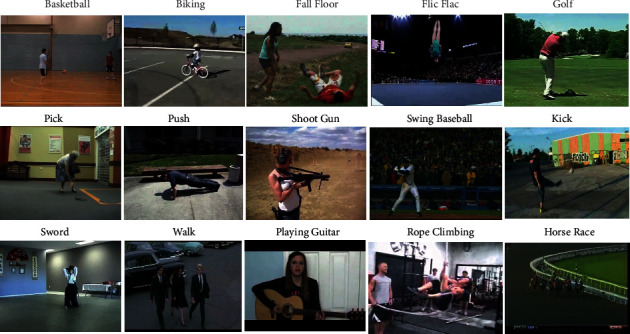
Sample action categories of UCF50 and HMDB51 datasets.

The performance of the proposed framework is validated over two benchmark datasets, UFC50 [39] and HMDB51 [40]; their visual samples are shown in Figure 3. The proposed method is implemented using Python (3.6 version) in Spyder integrated development environment.

A famous deep learning framework TensorFlow (2.5.0 version) with Keras backend and additional libraries including OpenCV, Scikit-image, and NumPy are used during experimentation. Furthermore, in the system configuration, Ubuntu operating system with GeForce RTX 2080-TI graphics card is used to accelerate the training process. The standard evaluation metrics such as Precision, Recall, F1-score, and Accuracy are used to evaluate the performance of the proposed method, as given in Table 4.

Table 5 shows the comparative results when compared to state-of-the-art models on UCF50 and HMDB51 datasets. In the subsequent sections, we define the datasets used in our experimentation along with discussion about our model's performance on the mentioned datasets.

### 3.1. UCF50

**Figure 4:**
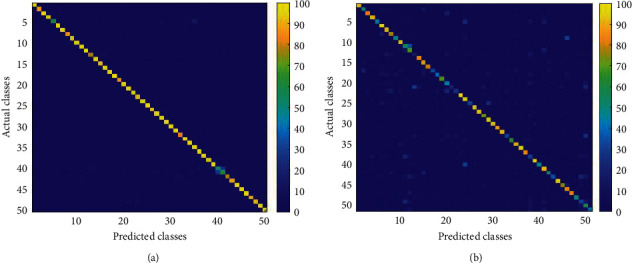
Confusion matrix of the proposed model. (a) UCF50 and (b) HMDB51 dataset.

**Figure 5:**
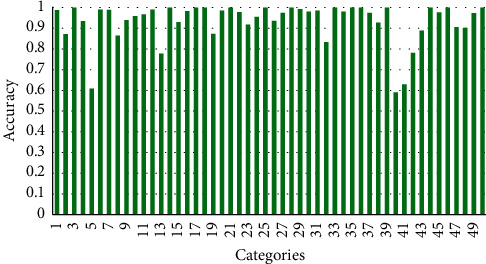
Class-wise accuracy of UCF50 dataset on the proposed ViT and multilayer LSTM model.

UCF50 is a very popular HAR dataset consisting a total of 50 classes; all the video clips are collected from YouTube in “.avi” format. Each class in the video is divided into different groups that share common features; for example, in one group, a piano is played by a person four times but with a different viewpoint. Furthermore, it consists of a diverse collection of human activities due to high diversity in the camera motion, poses, and object appearances, viewpoints, clutter background, and different illumination in the surroundings. The performance of the proposed method is compared with different state-of-the art methods, for example, Handcrafted, LSTM, and Non-LSTM-based methods, as given in Table 5, where the last row shows the accuracy of the proposed method in percentage. Confusion matrix is given in Figure 4(a), where we have achieved 96.144% accuracy. Class-wise accuracy is shown in Figure 5, where the accuracies of the majority of classes are higher than 90%. For the comparative analysis, we have evaluated the performance of the proposed method with improved dense trajectories (IDT) hybrid approach [42], achieving 92.3% accuracy, while the LSTM-based method, the temporal optical flow with multi-layer LSTM [47], and lightweight CNN with DS-GRU [21] have achieved the second highest accuracies of 94.9% and 95.2%, respectively.

### 3.2. HMDB51

**Figure 6:**
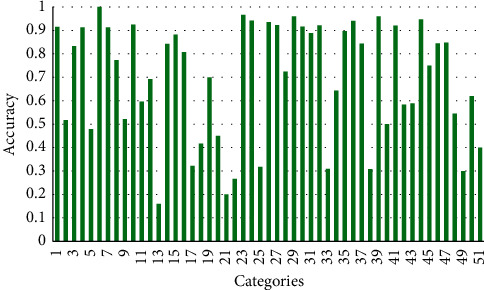
Class-wise accuracy of HMDB51 dataset on the proposed ViT and multilayer LSTM model.

The HMDB51 dataset contains different varieties of videos related to human body movements such as facial interaction, object interaction with body, and human interaction for body movements. There are 6766 action video clips collected from different unique sources, all the video clips belong to 51 classes. Most of the activities are less than five seconds duration with each video frame resized into 224×224 dimensions for the training purpose. This dataset is very challenging because all the video clips are collected in challenging environments such as different illumination; four to six video clips of the same class and subjects are recorded in different pose and viewing orientations. Table 5 shows the comparative analysis of the proposed method with existing state-of-the-art methods such Handcrafted, LSTM, and Non-LSTM methods. The confusion matrix is shown in Figure 4(b), where the highest true positive value of each category is represented along in the diagonal; our proposed method achieved 73.714% accuracy. The class-wise accuracy is shown in Figure 6, where the horizontal axis represents numbers of classes, and the vertical axis shows the percentage accuracy of the corresponding category.

To summarise the existing literature, the highest accuracies of 62.2%, 72.3%, and 57.2% are achieved by Handcrafted, CNN and LSTM, and Non-LSTM-based methods, respectively. Meanwhile our proposed method improves the performance up to 1.414% on HMDB51 dataset when compared to these models, as given in Table 5. Similarly, the class-wise accuracy is shown in Figure 6, where our method achieved the best accuracy against rivals.

## 4. Conclusions

An action is a sequence of multiple successive frames; thereby, both spatial and temporal features play an important role in accurate HAR. For this purpose, we have used pretrained ViT-Base-16 to extract the spatial features at predefined time stamps. These spatial features are fed to multilayered LSTM network to learn the long-range temporal dependencies. We have performed extensive experiments on two standard HAR datasets, UCF50 and IMDB51, and achieved recognition accuracies of 73.714% and 96.144%, respectively. However, in the proposed framework, there are few limitations that we will cover in the future research. For example, for HAR, we have used single-view camera that cannot provide full 360° coverage; in the future, we aim to use multiview data for efficient HAR. Moreover, the proposed framework is aimed to be transformed to an embedded platform to perform edge activity recognition. For efficient learning, two-stream networks with incremental learning strategy will be used to make it more intelligent to recognize complex actions in resource-constrained environments. Furthermore, in the future, we will combine different variants of ViT and different vision-based transformers models such as SWIN Transformer and ViViT for HAR. Our current system is flexible and can be helpful for adaptation in other domains such as emotion recognition, video summarization, and big data analytics.

## Figures and Tables

**Table 1:** Parameters details shown in the formulation of LSTM network.

Variables/symbol	Description
Δt	Input over time *t*
∂	Sigmoid activation function
w	Weights
b	Bias terms
i_t_	Input gate
f_t_	Forget gate
	Output gate
tanh	Tan *h* activation function
SoftMax	Activation for the final classification
N	Numbers of classes

**Table 2:** Different variants of ViT model used for image classification.

Model	Layers	Hidden size	MLP size	Heads	Params (M)
**ViT-Base**	12	768	3072	12	86
ViT-Large	24	1024	4096	16	307
ViT-Huge	32	1280	5120	16	632

The proposed method for features extraction is represented in bold text.

**Table 3:** The proposed LSTM network to capture long-range temporal information from video sequences.

Layer (type)	Output shape	No. of parameters
Input data	(None, 30, 1000)	0
LSTM	(None, 30, 128)	578048
LSTM	(None, 64)	49408
Dropout	(None, 64)	0
Batch normalization	(None, 64)	256
Activation	(None, 64)	0
Dense	(None, 64)	4160
Dense	(None, 51)	3315
Activation	(None, 51)	0

**Table 4:** Performance evaluation of the proposed method using Precision, Recall, and F1-score.

Dataset	Precision (%)	Recall (%)	F1-score (%)
UCF50	96.18655	96.14458	96.08283
HMDB51	76.49243	73.71429	73.51059

**Table 5:** Comparative analysis of the proposed method with traditional, deep LSTM, and non-LSTM-based techniques using HMDB51 and UCF50 datasets.

Domain	Technique	Accuracy (%)	
		HMDB51	UCF50
Handcrafted methods	Gradient boundary histogram + motion boundary descriptor [41]	62.2	--
	Improved dense trajectories (IDT) hybrid approach [42]	61.1	92.3
	Multiview super vector [43]	55.9	--
LSTM-based methods	Adoptive recurrent convolutional hybrid (ARCH) network [44]	58.2	--
	Lattice-LSTM [45]	66.2	--
	Relational LSTM [35]	71.4	--
	TS-LSTM and temporal inception [46]	69.0	--
	Temporal optical flow with multilayer LSTM [47]	72.2	94.9
	3D-CNNs and bidirectional hierarchical LSTM [48]	71.9	--
	CNN and DS-GRU [21]	72.3	95.2
Non-LSTM-based methods	Improved trajectory [49]	57.2	91.2
	Hierarchical clustering multitask learning [50]	51.4	93.2
The proposed method	ViT and multilayer LSTM	**73.714**	**96.144**

The methods represented by bold text show the highest performance in their respected categories.

## Data Availability

The codes and related materials can be downloaded from https://github.com/Altaf-hucn/ViTLSTM-HAR.
